# Genetically predicted circulating vitamin C in relation to cardiovascular disease

**DOI:** 10.1093/eurjpc/zwab081

**Published:** 2022-01-11

**Authors:** Shuai Yuan, Ju-Sheng Zheng, Amy M. Mason, Stephen Burgess, Susanna C. Larsson

**Affiliations:** 1 Unit of Cardiovascular and Nutritional Epidemiology, Institute of Environmental Medicine, Karolinska Institutet, Nobelsväg 13, Stockholm 17177, Sweden; 2 Westlake Laboratory of Life Sciences and Biomedicine, Shilongshan Road 18, Cloud Town, Xihu District, Hangzhou, China; 3 Key Laboratory of Growth Regulation and Translational Research of Zhejiang Province, School of Life Sciences, Westlake University, Shilongshan Road 18, Cloud Town, Xihu District, Hangzhou, China; 4 British Heart Foundation Cardiovascular Epidemiology Unit, Department of Public Health and Primary Care, University of Cambridge, Wort’s Causeway, Cambridge, CB1 8RN, UK; 5 National Institute for Health Research Cambridge Biomedical Research Centre, University of Cambridge and Cambridge University Hospitals, Hills Road, Cambridge CB2 0QQ, UK; 6 MRC Biostatistics Unit, University of Cambridge, East Forvie Building, Forvie Site, Robinson Way, Cambridge Biomedical Campus Cambridge, CB2 0SR, UK; 7 Department of Public Health and Primary Care, University of Cambridge, Strangeways Research Laboratory, Worts Causeway, Cambridge CB1 8RN, UK; 8 Unit of Medical Epidemiology, Department of Surgical Sciences, Uppsala University, Akademiska sjukhuset, ingång 78, 1tr, 751 85 Uppsala, Sweden

**Keywords:** Cardiovascular disease, Mendelian randomization, Vitamin C

## Abstract

**Aim:**

We conducted a two-sample Mendelian randomization (MR) study to assess the associations of genetically predicted circulating vitamin C levels with cardiovascular diseases (CVDs).

**Methods and results:**

Ten lead single-nucleotide polymorphisms associated with plasma vitamin C levels at the genome-wide significance level were used as instrumental variables. Summary-level data for 15 CVDs were obtained from corresponding genetic consortia, the UK Biobank study, and the FinnGen consortium. The inverse-variance-weighted method was the primary analysis method, supplemented by the weighted median and MR-Egger methods. Estimates for each CVD from different sources were combined. Genetically predicted vitamin C levels were not associated with any CVD after accounting for multiple testing. However, there were suggestive associations of higher genetically predicted vitamin C levels (per 1 standard deviation increase) with lower risk of cardioembolic stroke [odds ratio, 0.79; 95% confidence interval (CI), 0.64, 0.99; *P* = 0.038] and higher risk of atrial fibrillation (odds ratio, 1.09; 95% CI, 1.00, 1.18; P = 0.049) in the inverse-variance-weighted method and with lower risk of peripheral artery disease (odds ratio, 0.76, 95% CI, 0.62, 0.93; P = 0.009) in the weighted median method.

**Conclusion:**

We found limited evidence with MR techniques for an overall protective role of vitamin C in the primary prevention of CVD. The associations of vitamin C levels with cardioembolic stroke, atrial fibrillation, and peripheral artery disease need further study.

## Introduction

Vitamin C as an antioxidant has been proposed to alleviate oxidative stress and affect vascular remodelling, endothelial function, and lipid peroxidation, thereby potentially having a protective role in cardiovascular disease (CVD).^1–3^ Observational data have indicated that a high circulating level or intake of vitamin C is associated with a reduced risk of CVD and corresponding mortality.^4–8^ Several biological processes and signalling pathways have been proposed to be involved in the potential therapeutic effects of vitamin C on CVD.^9^ However, the cardio-protective effect of vitamin C has not been validated in randomized controlled trials (RCTs) of supplementation with vitamin C alone or together with other antioxidative vitamins.^3,10,11^ Thus, any causal relationship between vitamin C and CVD remains unestablished given potential confounding in previous observational findings and certain limitations of RCTs (e.g. a small sample size, imbalanced baseline characteristics, combined supplementation of vitamin C with other nutrients, and low compliance to intervention).

Utilizing genetic variants as instrumental variables for an exposure (e.g. plasma vitamin C levels) allows the Mendelian randomization (MR) design to more plausibly investigate causal inferences by minimizing residual confounding and other biases. Here, we conducted a two-sample MR study to assess the associations of genetically predicted circulating vitamin C levels with risk of a wide range of CVDs.

## Methods

### Outcome data sources

We included 15 cardiovascular endpoints with numbers of cases ranging from 3373 (large artery stroke) to 139 364 (coronary artery disease). Summary-level data for these outcomes were obtained from large genetic consortia,^12–16^ the UK Biobank study,^17^ and the FinnGen consortium.^18^ Detailed descriptions on data sources are presented in Table 1.

### Instrument selection

Eleven lead single-nucleotide polymorphisms (SNPs) associated with plasma vitamin C levels at the genome-wide significance level (*P*<5 x 10^−8^) were identified from a meta-analysis of genome-wide association studies (GWASs) including up to 52 018 individuals of European descent.^19^ These SNPs explained approximately 1.87% of variance in circulating vitamin C levels.^19^ Rs33972313 in the *SLC23A1* gene region encodes the sodium-dependent vitamin C transporter 1 and explained most of the variance in circulating vitamin C levels. One additional effect allele of this variant is associated with an 11% higher plasma vitamin C level.^20^ Rs174547 in *FADS1* gene region was excluded from analyses due to pleiotropic effects on plasma phospholipid fatty acids,^21^ leaving 10 SNPs leveraged as instrumental variables (Table 2). Rs13028225 was not available in FinnGen data of SNP-CVD associations and was replaced by rs17655123 in high linkage disequilibrium (*r*^2^ = 0.88) with rs13028225. Likewise, rs4074995 (*r*^2^ = 0.83) and rs3809260 (*r*^2^ = 0.98) were used as the proxy SNPs for rs10051765 and rs2559850, respectively, in the analysis of data from the International Stroke Genetics Consortium. Association estimates in the vitamin C GWAS were adjusted for age, sex, the first 10 genetic principal components, and study centre (where applicable).

### Statistical analysis

The multiplicative random-effects inverse-variance-weighted method^22^ was used as the main statistical method to assess the association between genetically predicted circulating vitamin C levels and CVDs. MR estimates for each CVD outcome from different data sources were combined using the fixed-effects meta-analysis method. We used two supplementary analyses, the weighed median approach^23^ and MR-Egger regression,^24^ to examine the robustness of the results and possible pleiotropy. The weighted median method can provide consistent causal estimates provided that ≥50% of the weight comes from valid SNPs.^23^ MR-Egger regression can detect horizontal pleiotropy by *P*-value for its intercept and generate estimate after correction for pleiotropy.^24^ The *I*^2^ statistic was calculated to assess the degree of heterogeneity^25^ among estimates of SNPs in each analysis. All reported odds ratios (ORs) and corresponding 95% confidence intervals (CIs) of CVDs were scaled to 1 standard deviation (SD) increase in genetically predicted circulating levels of vitamin C. The Bonferroni method was used to adjust for multiple testing (15 CVDs). Associations with P-values of <0.003 were regarded as significant associations and associations with P-values between 0.05 and 0.003 were deemed as suggestive associations. All analyses were two-sided and performed using the mrrobust package^26^ in Stata/SE 15.0.

## Results

**Figure 1:**
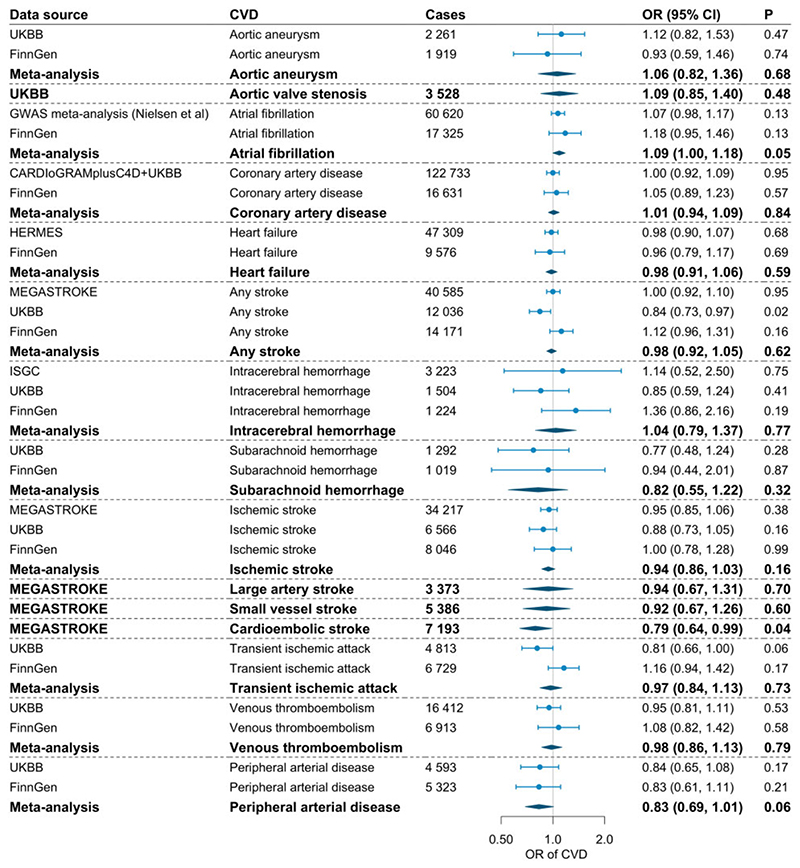
Associations of genetically predicted circulating vitamin C levels with risk of cardiovascular disease. CARDIoGRAMplusC4D, Coronary ARtery DIsease Genome-wide Replication and Meta-analysis plus The Coronary Artery Disease Genetics; CVD, cardiovascular disease; HERMES; Heart Failure Molecular Epidemiology for Therapeutic Targets; ISGC, International Stroke Genetic Consortium; LB, lower bound of 95% confidence interval; OR, odds ratio; UKBB, UK Biobank; UB, upper bound of 95% confidence interval. The UK Biobank was included in the GWAS meta-analysis for atrial fibrillation (Nielsen *et al*.), HERMES consortium, and ISGC.

The associations of genetically predicted circulating vitamin C levels (per 1 SD increase) with the 15 CVDs in the main analysis are presented in Figure 1. We observed suggestive inverse associations of genetically predicted vitamin C levels with risk of any stroke in UK Biobank (OR, 0.84; 95% CI, 0.73, 0.97; *P*= 0.018) and cardioembolic stroke in MEGASTROKE (OR, 0.79; 95% CI, 0.64, 0.99; *P* = 0.038). However, the association for any stroke was not replicated in the MEGASTROKE and FinnGen consortia and did not persist in the meta-analysis. There was no association between genetically predicted vitamin C and atrial fibrillation in the GWAS meta-analysis by Nielsen *et al*. or in the FinnGen consortium, but the meta-analysis results revealed a suggestive positive association (OR 1.09, 95% CI, 1.00, 1.18; *P*= 0.049). Genetically predicted vitamin C levels were not associated with the other studied CVDs in the main analysis.

Results of the supplementary analyses based on the weighted median and MR-Egger methods showed no association of genetically predicted vitamin C levels with any CVD (Table 3), with the exception for a suggestive inverse association with peripheral artery disease in the FinnGen consortium (OR, 0.71, 95% CI, 0.52, 0.97; *P* = 0.030) and the meta-analysis (OR, 0.76, 95% CI, 0.62, 0.93; *P* = 0.009). We observed the modest heterogeneity in several analyses; however, the P-values for the intercept in corresponding MR-Egger regression were >0.05 (Table 3). In a supplementary analysis using rs33972313 in the *SLC23A1* gene region as instrumental variable, the associations of genetically predicted vitamin C levels with peripheral artery disease (OR, 0.67, 95% CI, 0.46, 1.00; *P* = 0.048) and other CVDs (data not shown) were consistent with the findings from the analyses based on all instrumental variables.

## Discussion

**Figure 2:**
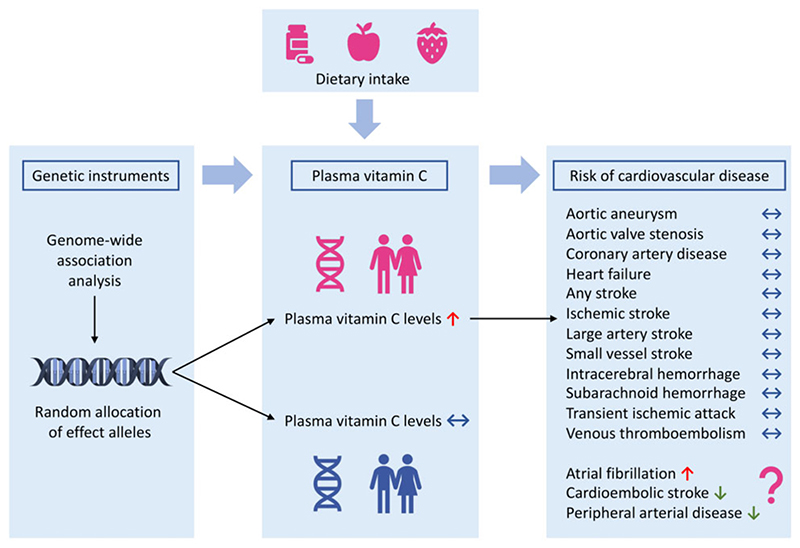
Summary of associations of genetically predicted plasma vitamin C levels with risk of cardiovascular disease.

This MR study found no clear pattern of associations between genetically predicted vitamin C levels and risk of CVDs (Figure 2). However, genetically predicted vitamin C levels showed suggestive inverse associations with cardioembolic stroke and peripheral artery disease but a suggestive positive association with atrial fibrillation (Figure 2).

The overall lack of support for a protective association between vitamin C and CVDs in the present MR study was consistent with most RCTs^3,10,11^ and some^27,28^ but not all^4–7^ observational studies. A recent review article concluded that findings differed between RCTs for vitamin C supplementation with null findings and observational studies on dietary vitamin C intake suggesting a protective association.^8^ Several signalling pathways were highlighted using the network pharmacology approach,^9^ whereas no evidence was found to support that vitamin C supplementation reduced the risk of CVD in healthy participants in a systematic review of RCTs.^10^ This discrepancy may be caused by residual confounding by other cardio-protective nutrients, such as magnesium,^29,30^ from foods rich in vitamin C (e.g. fruit and vegetables), or healthy lifestyle behaviours among individuals with a vitamin C-rich diet.^31^ Even though the present MR study did not support cardiovascular benefits of increasing circulating vitamin C levels, our findings did not hint any information on possible health benefits from a diet abundant in vitamin C, suggested by previous evidence.^4,32,33^ Instead, the present study did not justify vitamin C supplementation as a primary prevention strategy for CVD.

Higher plasma vitamin C levels were associated with a reduced risk of total stroke in cohort studies.^34^ Nevertheless, a daily supplementation of 500 mg of vitamin C together with 400 IU of vitamin E did not decrease the risk of total stroke in an RCT involving 14 641 US male physicians followed-up of for a mean of 8 years.^35^ Data on cardioembolic stroke are sparse. Likewise, few studies have investigated whether vitamin C status was associated with incident peripheral artery disease, although a clinical study revealed that acute vitamin C administration might restore peripheral endothelial function in patients with coronary artery disease.^36^ Plasma vitamin C was inversely associated with risk of atrial fibrillation in middle-aged women with low baseline intake but not in men^37^ and might prevent post-operative atrial fibrillation albeit with heterogeneous findings.^38^ Our study, on the contrary, found a possible positive association of genetically predicted circulating vitamin C levels with atrial fibrillation, a finding that needs to be verified in other studies.

A previous MR study found no association between plasma vitamin C proxied by rs33972313 in the *SLC23A1* gene region and ischaemic heart disease,^20^ which is consistent with our findings. In addition, genetically proxied plasma vitamin C was not associated with certain cardiovascular risk factors, such as type 2 diabetes^19^ and urate.^39^

The major strength of the present study is the MR design, which diminished residual confounding and other biases, thereby strengthening the causal inference. In addition, we examined the association of genetically predicted vitamin C levels with CVDs using several data sources and the consistency of results the consistency of results supports the robustness of our findings. Along with the use of multiple independent SNPs as instrumental variables for plasma vitamin C, combining results for one outcome from different data sources increased the statistical power to detect weak associations even though we might have overlooked associations for certain outcomes with small number of cases. We confined the analyses to individuals of European ancestry, with the exception for the analysis for coronary artery disease based on consortium data where >80% of participants are individual of European descent. Thus, our findings were less likely to be influenced by population structure bias. Nonetheless, the population confinement limited the generalizability of our findings.

A potential limitation in MR studies is horizontal pleiotropy. We excluded an SNP (rs174547) with pleiotropic effects in the analysis to minimize bias from horizontal pleiotropy. Results were broadly consistent across sensitivity analyses and no evidence of horizontal pleiotropy was detected by MR-Egger regression. We also examined the association of plasma vitamin C with CVD using rs33972313^20^ in the *SLC23A1* gene, which encodes the sodium-dependent vitamin C transporter 1, as instrumental variable and found consistent results. No evidence of bias from horizontal pleiotropy was detected. Another limitation is that we could not assess potential interaction effects of vitamin C with other antioxidants (e.g. vitamin E and β-carotene) on CVD. Our findings were based on generally healthy population and therefore cannot be generalized to populations with special features, such as individuals with vitamin C deficiency, patients with diabetes or kidney disease, and heavy smokers. There was small sample overlap in certain analyses. This overlap might have caused minor bias in the estimates towards the observational associations. Potential non-linear associations could not be examined in this MR study based on summary-level data.

In conclusion, this MR study suggests that elevating circulating vitamin C levels may not benefit the primary prevention of most CVDs. Whether increased vitamin C levels may decrease the risk of cardioembolic stroke and peripheral artery disease needs confirmation.

## Figures and Tables

**Table 1:** Information on outcome data sources

Data source	Cardiovascular disease	Cases	Controls	Population	Covariates adjusted in GWAS
GWAS meta-analysis (Nielsen *et al*.)	Atrial fibrillation	60 620	970 216	European	Birth year, sex, genotype batch, and 1-4 principal components
CARDIoGRAMplusC4D plus UKBB	Coronary artery disease	122 733	424 528	Mixed	NA
HERMES consortium	Heart failure	47 309	930 014	European	Age and sex, and principal components in individual studies where applicable
MEGASTROKE consortium	Stroke	40 585	406 111	European	Age and sex
	Ischaemic stroke	34 217	NA		
	Large artery stroke	3373	406 111		
	Small vessel stroke	5386	406 111		
	Cardioembolic stroke	7193	406 111		
ISGC	Intracerebral hemorrhage	3223	3725	European	Age, sex, and principal components
The UK Biobank study (UKBB)	Aortic aneurysm	2261	365 300	European	Age, sex, and 10 genetic principal components
	Aortic valve stenosis	3528	364 033		
	Stroke	12 036	355 525		
	Intracerebral hemorrhage	1504	366 057		
	Subarachnoid hemorrhage	1292	366 269		
	Ischaemic stroke	6566	360 995		
	Transient ischaemic attack	4813	362 748		
	Venous thromboembolism	16 412	351 149		
	Peripheral vessel disease	4593	362 968		
The FinnGen consortium	Aortic aneurysm	1919	167 843	European	Age, sex, the first 10 genetic principal components, and genotyping batch
	Atrial fibrillation	17 325	97 214		
	Coronary artery disease	16 631	160 268		
	Heart failure	9576	159 286		
	Stroke	14 171	133 027		
	Intracerebral hemorrhage	1224	163 533		
	Subarachnoid hemorrhage	1019	163 508		
	Ischaemic stroke	8046	164 286		
	Transient ischaemic attack	6729	164 286		
	Venous thromboembolism	6913	169 986		
	Peripheral vessel disease	5323	167 843		

CARDIoGRAMplusC4D, Coronary ARtery DIsease Genome-wide Replication and Meta-analysis plus The Coronary Artery Disease Genetics; GWAS, genome-wide association study; HERMES; Heart Failure Molecular Epidemiology for Therapeutic Targets; ISGC, International Stroke Genetic Consortium; NA, not available. The UK Biobank was included in Consortium (Nielsen et *al*.), HERMES consortium, and ISGC.

**Table 2:** Information on instrumental variables

SNP	Chr	Position	Nearby gene	EA	NEA	EAF	Beta	SE	P-value
rs6693447	1	2330190	RĒR1	T	G	0.551	0.039	0.006	6.25E-10
rs13028225	2	220031255	*SLC23A3*	T	C	0.857	0.102	0.009	2.38E-30
rs33972313	5	138715502	*SLC23A1*	C	T	0.968	0.360	0.018	4.61E-90
rs10051765	5	176799992	*RGS14*	C	T	0.342	0.039	0.007	3.64E-09
rs7740812	6	52725787	*GSTA5*	G	A	0.594	0.038	0.006	1.88E-09
rs117885456	12	96249111	*SNRPF*	A	G	0.087	0.078	0.012	1.70E-11
rs2559850	12	102093459	*CHPT1*	A	G	0.598	0.058	0.006	6.30E-20
rs10136000	14	105253581	*AKT1*	A	G	0.283	0.040	0.007	1.33E-08
rs56738967	16	79740541	*MAF*	C	G	0.321	0.041	0.007	7.62E-10
rs9895661	17	59456589	*BCAS3*	T	C	0.817	0.063	0.008	1.05E-14

Chr, chromosome; EA, effect allele; EAF, effect allele frequency; NEA, non-effect allele; SE, standard error; SNP, single-nucleotide polymorphism.

**Table 3:** Supplementary analyses of the associations of genetically predicted circulating vitamin C with cardiovascular disease

Data source	Cardiovascular disease	Cases	Controls	*I*^2^ (%)	Weighted median method			MR-Egger regression			
					OR	95% CI	*P*	OR	95% CI	*P*	*P_intercept_*
UKBB	Aortic aneurysm	2261	365 330	0	1.05	0.71, 1.56	0.801	0.96	0.58, 1.59	0.880	0.443
FinnGen	Aortic aneurysm	1919	167 843	30	0.80	0.48, 1.33	0.384	0.57	0.28, 1.17	0.126	0.105
UKBB	Aortic valve stenosis	3528	364 033	0	1.12	0.82, 1.53	0.482	1.32	0.89, 1.94	0.166	0.226
GWAS meta-analysis (Nielsen et *al.)*	Atrial fibrillation	60 620	970 216	0	1.07	0.97, 1.17	0.161	1.08	0.93, 1.26	0.307	0.860
FinnGen	Atrial fibrillation	17 325	97 214	29	1.11	0.87, 1.41	0.413	1.13	0.76, 1.67	0.540	0.784
CARDIoGRAMplusC4D + UKBB	Coronary artery disease	122 733	424 528	30	1.00	0.91, 1.10	0.982	0.99	0.86, 1.14	0.932	0.954
FinnGen	Coronary artery disease	16 631	160 268	0	0.95	0.77, 1.18	0.647	0.81	0.61, 1.07	0.135	0.025
HERMES	Heart failure	47 309	930 014	0	1.02	0.92, 1.13	0.771	1.07	0.94, 1.23	0.308	0.101
FinnGen	Heart failure	9576	159 286	11	0.90	0.71, 1.15	0.415	0.82	0.58, 1.14	0.233	0.242
MEGASTROKE	Stroke	40 585	406 111	5	0.99	0.88, 1.10	0.810	0.94	0.81, 1.09	0.397	0.265
UKBB	Stroke	12 036	355 525	9	0.90	0.75, 1.07	0.241	0.89	0.71, 1.12	0.331	0.543
FinnGen	Stroke	14 171	133 027	0	1.15	0.92, 1.42	0.217	1.11	0.83, 1.47	0.487	0.913
ISGC	Intracerebral hemorrhage	3223	3725	47	0.96	0.46, 2.04	0.925	0.69	0.19, 2.43	0.560	0.318
UKBB	Intracerebral hemorrhage	1504	366 057	0	0.75	0.46, 1.21	0.237	0.83	0.46, 1.49	0.528	0.887
FinnGen	Intracerebral hemorrhage	1224	163 533	0	2.08	1.10, 3.92	0.024	2.08	0.94, 4.61	0.071	0.202
UKBB	Subarachnoid hemorrhage	1292	366 269	26	0.76	0.46, 1.28	0.309	0.83	0.38, 1.81	0.634	0.828
FinnGen	Subarachnoid hemorrhage	1019	163 508	56	1.00	0.47, 2.14	0.999	2.01	0.59, 6.86	0.265	0.137
MEGASTROKE	Ischaemic stroke	34 217	NA	0	0.96	0.83, 1.11	0.553	0.90	0.75, 1.08	0.270	0.477
UKBB	Ischaemic stroke	6566	360 995	0	0.87	0.68, 1.10	0.248	1.00	0.75, 1.33	0.991	0.239
FinnGen	Ischaemic stroke	8046	164 286	41	1.05	0.81, 1.37	0.712	1.15	0.75, 1.77	0.523	0.428
MEGASTROKE	Large artery stroke	3373	406 111	28	0.95	0.65, 1.38	0.770	0.91	0.49, 1.66	0.748	0.896
MEGASTROKE	Small vessel stroke	5386	406 111	34	0.77	0.55, 1.07	0.114	0.67	0.42, 1.07	0.093	0.093
MEGASTROKE	Cardioembolic stroke	7193	406 111	0	0.83	0.63, 1.10	0.191	0.81	0.56, 1.18	0.269	0.883
UKBB	Transient ischaemic attack	4813	362 748	0	0.79	0.60, 1.03	0.076	0.78	0.56, 1.08	0.136	0.727
FinnGen	Transient ischaemic attack	6729	164 286	0	1.15	0.88, 1.50	0.313	1.09	0.76, 1.57	0.648	0.684
UKBB	Venous thromboembolism	16 412	351 149	46	1.01	0.87, 1.17	0.903	1.02	0.79, 1.32	0.900	0.504
FinnGen	Venous thromboembolism	6913	169 986	46	1.02	0.78, 1.34	0.875	0.93	0.57, 1.52	0.784	0.471
UKBB	Peripheral arterial disease	4593	362 968	25	0.80	0.61, 1.05	0.112	0.85	0.56, 1.29	0.445	0.936
FinnGen	Peripheral arterial disease	5323	167 843	38	0.71	0.52, 0.97	0.030	0.63	0.38, 1.03	0.063	0.178

CARDIoGRAMplusC4D, Coronary ARtery DIsease Genome-wide Replication and Meta-analysis plus The Coronary Artery Disease Genetics; CI, confidence interval; CVD, cardiovascular disease; HERMES; Heart Failure Molecular Epidemiology for Therapeutic Targets; ISGC, International Stroke Genetic Consortium; OR, odds ratio; SNP, single-nucleotide polymorphism; UKBB, UK Biobank. The *I*^2^ statistic was used to present the heterogeneity among estimates for each SNP in one analysis. The P-value for the intercept in the MR-Egger regression was used present the pleiotropy (*P* < 0.05). The UK Biobank was included in GWAS meta-analysis (Nielsen et *al*.), HERMES consortium, and ISGC.

## Data Availability

All data analysed in this study are available OSF data respiratory (https://osf.io/6qd8f/).
